# Complete mitochondrial genome of a malaria vector mosquito *Anopheles sinensis* from South Korea

**DOI:** 10.1080/23802359.2022.2077665

**Published:** 2022-05-26

**Authors:** Ashraf Akintayo Akintola, Bia Park, Eun Hwa Choi, Ui Wook Hwang

**Affiliations:** a School of Industrial Technology Advances, Kyungpook National University, Daegu, South Korea; b Department of Biology Education, Teachers College & Institute for Phylogenomics and Evolution, Kyungpook National University, Daegu, South Korea; c Institute for Korean Herb-Bio Convergence Promotion, Kyungpook National University, Daegu, South Korea

**Keywords:** *Anopheles sinensis*, mitochondrial genome, phylogeny, South Korea

## Abstract

We present the complete mitochondrial genome of a Malaria vector Mosquito *Anopheles sinensis* Wiedemann, 1828 from South Korea. The mitochondrial genome is about 15,421 bp long and contains 13 protein-coding genes (PCGs), 22 tRNA genes, two rRNA genes, and an A-T rich region. The present data were compared with those from China with respect to PCG sequence differences, tRNA structure, gene order, and control region (CR) structure. *An. sinensis* mitochondrial genomes from northeast Asia share identical gene composition and gene order. In contrast, they have distinct differences in the CR within the range 8.75% (51/583 bp) to 9.95% (58/583 bp). The phylogenetic analysis showed that *An. sinensis* from South Korea was clustered together with those from China, but there existed distinct genetic distance between the two. Likewise, mitochondrial genome sequences from other *Anopheles* species were employed to infer phylogenetic relationships among the members of the genus *Anopheles*. This study further promotes the enrichment of *An. sinensis* mitochondrial genome data, providing useful information for their mitochondrial genetic differences along with geographical distances in northeast Asia.

*Anopheles sinensis* Wiedemann, 1828 is an oriental species widely distributed in South Korea, China, and Japan as well as in other North and Southeast Asian Countries (Lu 1997; Hwang 2007; Shin 2014). It belongs to the *hyrcanus* group (subgenus *Anopheles*) which is a complex species assemblage that includes more than 20 closely related species in South Korea and China (Lu 1997; Hwang et al. 2004, 2006; Ma and Xu 2005, 2015; Ree et al. 2005). It is a vector of *Plasmodium vivax* and certain worms such as *Brugia malayi* that causes lymphatic filariasis (Hasegawa 1913; Zhang et al. 1991; Ree and Hwang 2000; Zhou et al. 2012). Despite its disputable malaria vector capacity, *An. sinensis* is still incriminated as a competent vector for *P. vivax* malaria due to its abundant population size and wide distribution, which have led to occasional local malaria epidemics or outbreaks throughout history (Chai et al. 1994; Cho et al. 1994; Kho et al. 2001; Ree et al. 2001; Ren et al. 2015). Due to its location on a peninsula, *An. sinensis* populations in South Korea may not be affected by the gene flow from the continent or nearby islands, thus, making the populations suitable for studying genetic diversity (Jung et al. 2007). Likewise, analysis of the full mitochondrial genome from the Northeast Asian population would be appropriate to analyze differences in each sample collection (Hwang and Kim 1999). In this study, we first attempted to characterize the complete mitochondrial genome of *An. sinensis* collected from South Korea, which was then compared with Chinese ones.

*An. sinensis* samples were collected by using light traps in the high-risk areas of malaria, Mungi-ri, Tanhyoen-myeon, Paju-si, Kyonggi-do, South Korea (GPS. N37°49′54.72″, E126°43′24.7″). The specimen is kept in Kyungpook National University (KNU), Daegu, South Korea under the voucher no. LEGOA050001 (collector: Ui Wook Hwang, uwhwang1@gmail.com) and total genomic DNA was isolated from tissues using a DNeasy Blood and Tissue Kit (Qiagen Co., Hilden, Germany) following the manufacturer’s protocol. The mitochondrial genome was amplified by the standard PCR method using the methods described by Luo et al. (2016). The complete mitochondrial genome was amplified using 18 primer pairs designed by Zhang et al. (2013). The amplified PCR products were checked on 1.0% agarose gel and purified using a QIAquick PCR Purification Kit (Qiagen Co., Hilden, Germany). Then, the amplicons were sequenced directly using an ABI Prism 3730 DNA sequencer (PerkinElmer, Waltham, MA) with a BigDye Termination Sequencing Kit. All alignments were performed in the Clustal X2 program (Larkin et al. 2007) and BioEdit 7.0.9 program (Hall 1999). The characterization of the protein-coding genes (PCGs), rRNAs, tRNAs, and control region (CR) was done using NCBI Basic Local Alignment Search Tool (BLAST) and tRNAscan-SE program (Chan and Lowe 2019). The PCG of the amino acid sequences used for the phylogenetic analysis was also aligned with Clustal X2 (Larkin et al. 2007). Phylogenetic relationships were inferred using the maximum-likelihood (ML) with IQ-TREE online webserver (Trifinopoulos et al. 2016). The phylogenetic tree was built with 1000 bootstrap replicates and substitution model mtART + F+I + G4.

The complete mitochondrial genome of *An. sinensis* (GenBank accession number: OK458560) is a double-stranded circular genome of 15,421 bp in size, containing 13 PCGs, 22 tRNA genes, two rRNA genes, and a CR. The whole nucleotide composition is 40.0% for A, 37.9% for T, 9.5% for G, and 12.7% for C, respectively, presenting an obvious A + T bias (77.9%). Most of genes of the mitochondrial genome are encoded on heavy strand except for four PCGs (*ND1*, *ND4*, *ND4L*, and *ND5*), nine tRNA genes (*trnQ*, *trnC*, *trnY*, *trnS*, *trnF*, *trnH*, *trnP*, *trnL*, and *trnV*), and two rRNA genes (*rrnL* and *rrnS*). Except for *COI*, *ND4L*, and *ND1* starting with TCG, CTT, and CAT, respectively, other PCGs use ATG, ATA, or ATC as the initial codon. Seven PCGs (*ND2*, *COI*, *APT8*, *ATP6*, *ND3*, *ND5*, and *ND4*) stop with the complete termination codon TAA, and the rest have incomplete stop codon T. All tRNAs vary from 66 to 72 bp in length. The overall A + T content of 22 tRNA genes is 78.5%. Two rRNA genes were found: *16S rRNA* with a length of 1268 bp and *12S rRNA* with a length of 756 bp. The *16S rRNA* is assumed to fill up the blanks between *trnL* and *trnV*, and the *12S rRNA* is located between *trnV* and the CR. The CR is 583 bp long which has a higher A + T content (93.7%) than that of the whole mitochondrial genome (78.9%).

To determine the phylogenetic position of *An. sinensis* from South Korea within the *Hyrcanus* group, we reconstructed the ML tree based on the concatenated amino acid sequence alignment deduced from eight *Anopheles* mitochondrial genomes. We employed *An. darlingi* as an outgroup. As shown in Figure 1, the major clades on the ML tree supported the monophylies of the two series *Myzorhynchus* (BP 98%) and *Anopheles* (BP 100%). *An. sinensis* from South Korea was clustered with Chinese ones, but there exhibited significant genetic distance between the two. Although *An. anthropophagus* appeared as a sister taxon of the monophyletic clade of *An. sinensis*, the two species are distinctly separated from each other.

**Figure 1.**
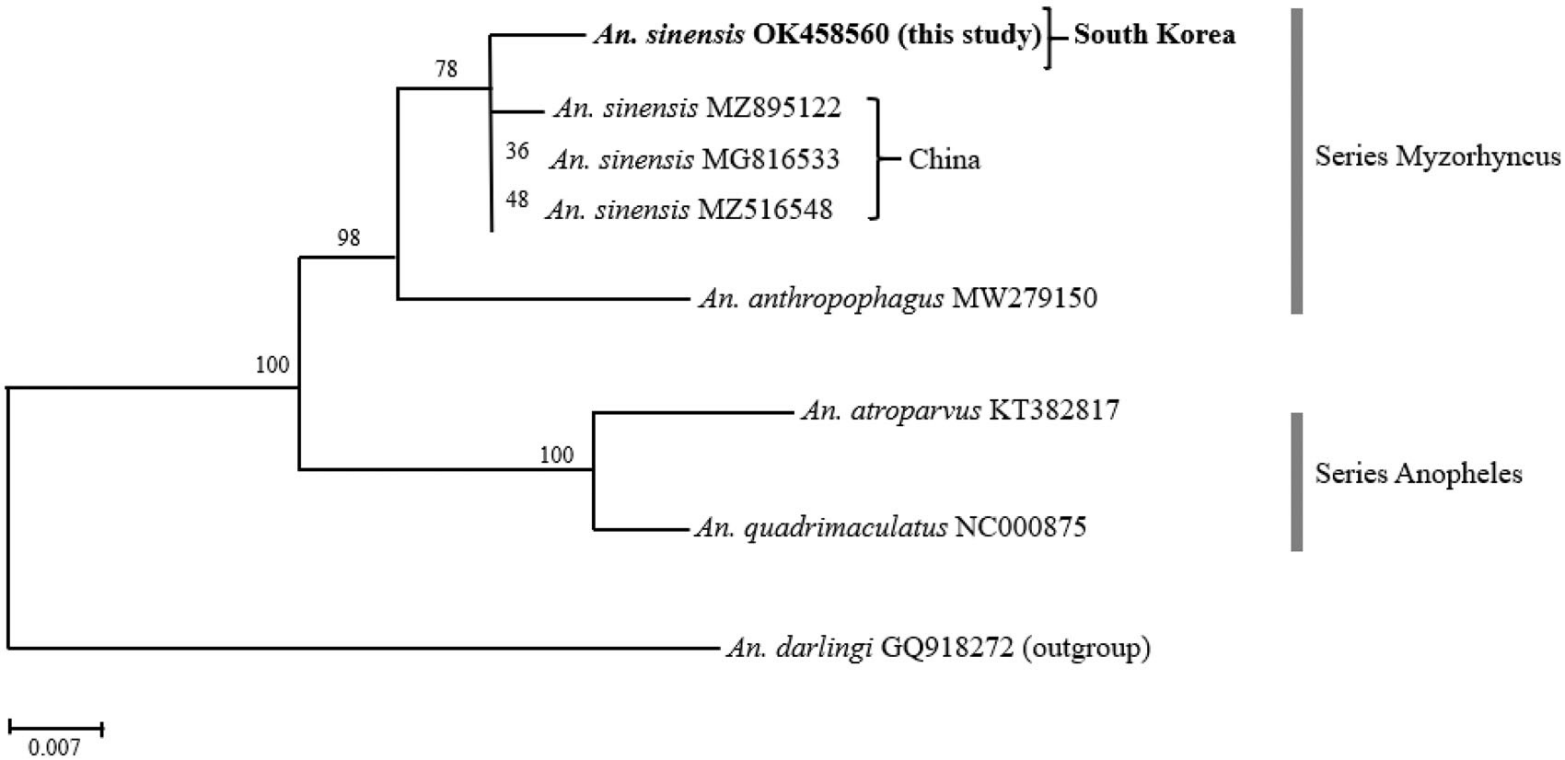
A maximum-likelihood (ML) tree showing the mitochondrial genetic distance between South Korean and Chinese *An. sinensis*. The ML tree was reconstructed based on amino acid sequences of the 13 mitochondrial PCGs under the mtART + F+I + G4 model using the IQTREE program. A newly sequenced *An. sinensis* individual from South Korea was highlighted in bold.

## Ethical approval

No specific permits were required for this study. The study did not involve endangered or protected species. Therefore, the ethics committee (Ministry of Environment, South Korea) deemed that approval was unnecessary.

## Author contributions

Ashraf Akintayo Akintola conceived and designed the experiments, performed the experiments, analyzed the data, contributed reagents/materials/analysis tools, prepared a figure, wrote the original draft, reviewed, and edited drafts of the manuscript. The experiments and data analysis were devised by Bia Park and Eun Hwa Choi, who then corrected critical drafts of the publication. Ui Wook Hwang conceived and designed the experiments and data analysis, reviewed and edited drafts of the manuscript approved the final draft.

## Data Availability

The complete mitochondrial genome sequence of *Anopheles sinensis* from South Korea has been deposited in GenBank and openly available in GenBank of NCBI at https://www.ncbi.nlm.nih.gov/nuccore/OK458560 under the accession no. OK458560. The associated BioProject, SRA, and Bio-Sample numbers are PRJNA823808, SRR18650725, and SAMN27361716, respectively.
